# Mites (Acari, Mesostigmata) in boreal Scots pine forest floors: effect of distance to stumps

**DOI:** 10.1007/s10493-014-9825-8

**Published:** 2014-06-05

**Authors:** Jacek Kamczyc, Dariusz J. Gwiazdowicz, Ewa Teodorowicz, Katarzyna Strzymińska

**Affiliations:** 1 Department of Game Management and Forest Protection, University of Life Sciences, Wojska Polskiego 71C, 60-625 Poznan, Poland; 2 Department of Forest Pathology, University of Life Sciences, Wojska Polskiego 71C, 60-625 Poznan, Poland

**Keywords:** Biodiversity, Coarse woody debris, CWD, Mites, Boreal forests, *Pinus sylvestris*

## Abstract

Coarse woody debris (CWD) is a basic component of forest ecosystems and it plays a crucial role in species-poor boreal forests. Generally, previous studies have focused on differences between the forest floor and decaying logs of various tree species. The impact of distance to CWD has been investigated mainly for forest-floor snails and some groups of macrofauna, but not yet for mesostigmatid mites communities. We hypothesized that the effect of CWD decreases with increasing distance from CWD. To test this hypothesis we conducted a study in relatively species-poor Finnish boreal forest (at ca. 100 km northwest of Helsinki). In total, 81 samples were collected in 2007 from nine Scots pine (*Pinus sylvestris*) stumps, three microhabitats (CWD, soil/litter at 0.5 m from a stump and soil/litter at 1.5 m from a stump) and in three main directions (9 stumps × 3 microhabitats × 3 directions). Overall, 1965 mesostigmatid mites were collected representing 24 species. The mean number of mite species collected was significantly different between decaying stumps and forest litter; however, there was no significant difference between the litter samples at 0.5 and 1.5 m distance. The evenness index was significantly lower for samples collected from stumps than for litter in close (0.5 m) or far (1.5 m) distance. The most frequently encountered mite species were *Veigaia nemorensis*, *Parazercon radiatus* and *Zercon zelawaiensis*.

## Introduction

Boreal forests are generally considered to be species-poor ecosystem when compared to tropical forests (Martikainen et al. 2000). Boreal forests were profoundly affected during the 1900s by large-scale intensive forestry in northern areas. This impact, which is clearly visible in Fennoscandia, Russia and Canada (Syrjanen et al. 1996; Bryant et al. 1997), can lead to ecological changes at the landscape as well as the local stand scale (Niemelä 1999). The changes are mostly due to the loss and fragmentation of old-growth forests, alternation of the structural components, spatial patterns and processes that are typical for natural forests (Similä et al. 2003). One of the most important factors that can locally enhance the species diversity and provides a habitat connectivity in forest ecosystems is coarse woody debris (CWD) (Kappes et al. 2009). Moreover, CWD may also improve arthropod diversity conservation, especially in managed forests (Castro and Wise 2010). The importance of CWD for nutrient turnover and for conservation on saproxylic (depending of dead wood) organisms is well established (Siira-Pietikäinen et al. 2008).

Generally, recent studies focused on the differences in abundance and species richness between CWD (i.e. fallen logs) and adjacent litter. They were conducted in temperate broad-leaved forests such as beech, oak, oak-beech forests (Jabin et al. 2004; Skubała and Duras 2008; Kappes et al. 2009) and in fir, pine, spruce and birch boreal forests (Setäla et al. 1995; Siira-Pietikäinen et al. 2008). Those studies documented the positive effect of CWD on abundance and diversity of nematodes, oribatids and collembolans and additionally suggest that the reduction of CWD may affect animal communities in litter. Much less information exists on the impact of distance to CWD on the mite community living in litter. The influence of close and far distance to CWD (i.e. moderately decayed logs) has been documented only for soil macroarthropods (Jabin et al. 2004; Castro and Wise 2010); no studies on microarthropods including mites have been conducted.

Our study focuses on mites (Acari: Mesostigmata) for several reasons. Mesostigmatid mites are still a poorly studied group in the CWD microhabitat. These mites have been well investigated only in four classes of decaying Scots pine (*Pinus* *sylvestris* L.) logs, located in pine-oak forest (Gwiazdowicz et al. 2011). However, that study did not include litter sampling. Additionally, mesostigmatid mites are among the most abundant groups in forest floors and they were also recorded in faunal studies from various types of decaying wood such as trunks, branches and stumps, where they occur at highest diversity (Karg 1993; Wiśniewski and Hirschmann 1993). Some species can colonize dead standing trees with bark beetles via phoresy, or actively from the forest litter when the tree falls down (Błoszyk 1999). Moreover, these mites play a crucial role in decomposition, since they can transport spores of fungi on their bodies into the decaying wood. Many of these fungi species are primary agents of wood decay in terrestrial forest ecosystems and contribute to log fragmentation (Barker 2008).

Therefore, we conducted a field study on Scots pine (*P.* *sylvestris*) stumps in relatively species-poor boreal forests and in litter in close (0.5 m) and far (1.5 m) distance to the ‘woody island’. We examined whether species richness differs between decaying wood (i.e. stumps) and soil/litter with increasing distance to the decaying wood. We tested the hypothesis that (1) mesostigmatid mite communities in Scots pine stumps differed from those in the forest floor in boreal forests, and (2) the distance to the ‘woody island’ affects these communities.

## Materials and methods

### Study site and sampling

**Fig. 1:**
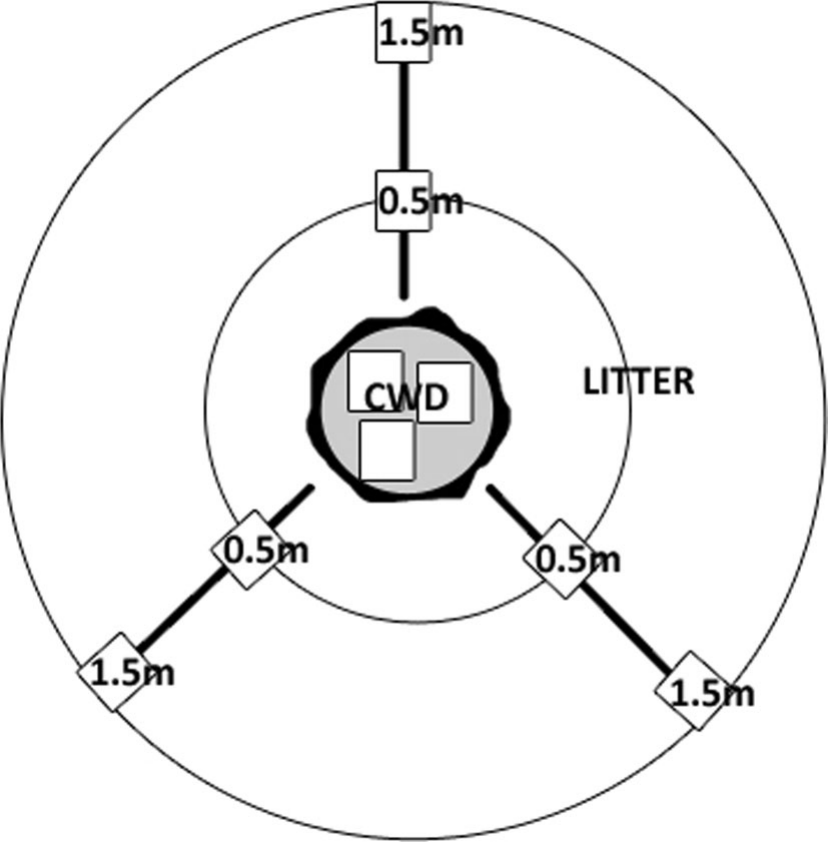
Sampling design in boreal Scots pine forests. Three samples were taken in stumps (i.e., coarse woody debris, CWD) and in litter/soil at 0.5 or 1.5 m distance from the stumps

Samples of decayed wood and litter were collected from Scots pine (*P. sylvestris*) forest close to the MTT Agrifood Research Finland, ca. 100 km northwest of Helsinki (60°49′N, 23°28′E). Forest floor microarthropods sampling was conducted once, on the same day, from the stump and litter in August 2007. The microhabitat conditions were similar, the ground was covered by the same plant/herbs species. The stand was mature (ca. 110 years old) and was classified as *Cladonio*-*Pinetum* association. Nine Scots pine stumps were selected (diameter 22–25 cm). Ground vegetation was dominated by *Cladonia rangiferina*, *Cladonia sylvatica*, *Corynephorus canescens* and *Festuca ovina.* The ‘nearest neighbour’ distance between stumps ranged from 15 to 25 m. In total, we collected 81 samples from nine tree stumps and from surrounding soil and litter. From each stump was taken: three samples of decaying wood directly from the stump, three samples from soil-litter at 0.5 m from the stump and three samples from soil-litter at 1.5 m from the stump, going in three directions (ca. 120° in between) (Fig. 1). These distances were set up to maintain homogeneity of the forest floor and to avoid edge effects of other types of litter microhabitats or live trees. The woody samples were carefully collected using a knife from the upper part of the stump, the core size was similar to samples collected from litter with a steel core (5 × 5 × 5 cm). Stumps were in the fourth class of wood decay, substantially decayed and pieces easily sloughed off. Inner heartwood was soft but intact. The outer surface was covered with mosses (Vanderwel et al. 2006).

Mites were extracted using Berlese funnels with a mesh size of approx. 2 mm where a temperature and moisture gradient forced active soil fauna to move down the core into 70 % ethanol over a period of 7 days. The total number of mesostigmatid mites was determined using a microscope. Mesostigmatid mites were mounted in permanent slides (using Hoyer’s medium) and semi-permanent slides (using lactic acid), and identified using universally applied keys such as Micherdziński (1969), Ghilarov and Bregetova (1977) and Karg (1993).

### Data analysis

Each soil/litter and CWD core provided an estimate of local (point) diversity and abundance and finally produces one data point for the statystical analysis. Diversity for each sample was measured using Shannon’s diversity index (*H’* = −Σ*p*_*i*_ ln *p*_*i*_, where *p*_*i*_ is the proportion of individuals found in the *i*-th species) and Eveness index (*E* = *H’*/ln [Richness]). Species richness was examined by counting the species in each sample. Species rank graph was restricted to the species representing at least 5 % of all individuals collected (dominance, *D*, ≥5 %). Abundance, eveness and species richness were statistically analysed with non-parametric Kruskal–Wallis tests, because variances were not homogeneous and data were not normally distributed. To test for significant differences in the Shannon index we used an ANOVA in SigmaPlot. Means were compared with Tukey’s post hoc test.

Theoretical total species richness for CWD and soil/litter in close and far distance was calculated using first- and second-order Jackknife estimates and Chao 1 and 2 estimates. All estimates were performed using EstimateSWin 8.20 (Colwell 2009). Chao 1 and 2, like the Jackknife estimate, are non-parametric methods for estimating species richness (Chao 1987). Chao 1 is based on the number of rare species (singletons and doubletons), whereas Chao 2 is based on presence/absence data. The Jackknife and Chao estimates become independent of sample size after half the theoretical total fauna is observed (Jackknife) or when the observed number of species is greater than the square root of 2× the theoretical total fauna (Chao) (Colwell and Coddington 1994).

The site pattern diversity of mite community assemblages was estimated using community similarity indices. Three distance measures were used: (1) Jaccard distance, based on the dissimilarity of species composition in paired samples; (2) Sørensen distance, based on the dissimilarity of species composition (presence/absence of species) in paired samples; and (3) Bray-Curtis distance, based on the dissimilarity of relative abundance in paired samples. Distances were calculated in EstimateSWin 8.20 (Colwell 2009). Species accumulation curves were based on the observed data.

Correspondence analysis (CA) was used to determine how species respond to various microhabitats (litter at close and far distance, CWD). The analysis was conducted using STATISTICA 10.0 (StatSoft, Tulsa, OK, USA) including all species in three types of microhabitat. Zoocenological analysis of mesostigmatid mite communities was based on indexes of dominance (D) and frequency (F) as described in Błoszyk (1999). The frequency was calculated as the percentage of samples in which the species was present. Dominance classes were used as follows: eudominants (>30 %), dominants (15.01–30 %), subdominants (7.01–15 %), residents (3.01–7 %) and subresidents (<3 %). Frequency classes were as follows: euconstants (>50 %), constants (30.01–50 %), subconstants (15.01–30 %), accessory species (5.01–15 %) and accidentals (<5 %).

## Results

### Number of species per sample and diversity index

In total, 1965 mesostigmatid mites were collected representing 24 species. Overall, the total number of species was highest in CWD (21) and lowest in litter at far distance (14). The mean number of species was significantly higher in CWD (9.2 ± 0.4) than in soil/litter at close (6.6 ± 0.3) or far distance (6.3 ± 0.3; χ^2^ = 28.63, df = 2, *P* < 0.001) (Table 1). Furthermore, mean abundance differed significantly between CWD and distant litter samples (χ^2^ = 33.31, df = 2, *P* < 0.001). The highest values were recorded in CWD (32.9 ± 2.6), the lowest in soil/litter at close distance (19.1 ± 0.6) (Table 1).

**Table 1:** Diversity of mesostigmatid mites (mean ± SEM) in decayed Scots pine tree stumps (coarse woody debris, CWD) and soil/litter at 0.5 and 1.5 m from the stumps

	CWD (stump)	Close (0.5 m)	Far (1.5 m)
Total number of species	21	16	14
Total abundance	861	465	639
Mean number of species	9.15 ± 0.42 a	6.55 ± 0.26 b	6.30 ± 0.25 b
Mean abundance	32.89 ± 2.57 a	19.07 ± 0.59 c	23.37 ± 1.20 b
Eveness (*E*)	0.73 ± 0.02 b	0.81 ± 0.01 a	0.79 ± 0.01 a
Shannon (*H’*)	1.56 ± 0.02 a	1.49 ± 0.02 ab	1.44 ± 0.03 b

Means within a row followed by the same letter are not significantly different (Tukey’s post hoc test: *P* < 0.05)

Generally, the diversity (*H’*) and eveness (*E*) of mesostigmatid mites differed among microhabitats (Table 1). Diversity was highest in CWD (1.6 ± 0.02) and lowest in litter at far distance (1.4 ± 0.03), whereas evenness was highest in litter at close distance (0.8 ± 0.01) (Table 1).

The rate of mesostigmatid species turnover among samples varied between microhabitats: species turnover in presence/absence (Jaccard and Sørensen distance) differed significantly among microhabitats and was highest for CWD and lowest for soil/litter at close distance to CWD (Table 2). The analysis of the Bray-Curtis dissimilarity (based on relative abundance) indicated significant differences between soil/litter at close versus far distance, but they both did not differ significantly from CWD (Table 2).

**Table 2:** Index of dissimilarity among samples (mean ± SEM) within habitat type: coarse woody debris (CWD) in decayed Scots pine tree stumps and soil/litter at 0.5 and 1.5 m from the stumps

Microhabitat	Jaccard distance	Sørensen distance	Bray-Curtis distance
CWD (stump)	0.56 ± 0.011 a	0.70 ± 0.009 a	0.54 ± 0.010 ab
Close (0.5 m)	0.43 ± 0.010 c	0.58 ± 0.009 c	0.49 ± 0.010 b
Far (1.5 m)	0.50 ± 0.011 b	0.64 ± 0.009 b	0.55 ± 0.011 a
*P* (ANOVA)	<0.001	<0.001	<0.001

Means within a column followed by the same letter are not significantly different (Tukey’s post hoc test: *P* < 0.05)

### Total species richness and species accumulation curves

**Fig. 2:**
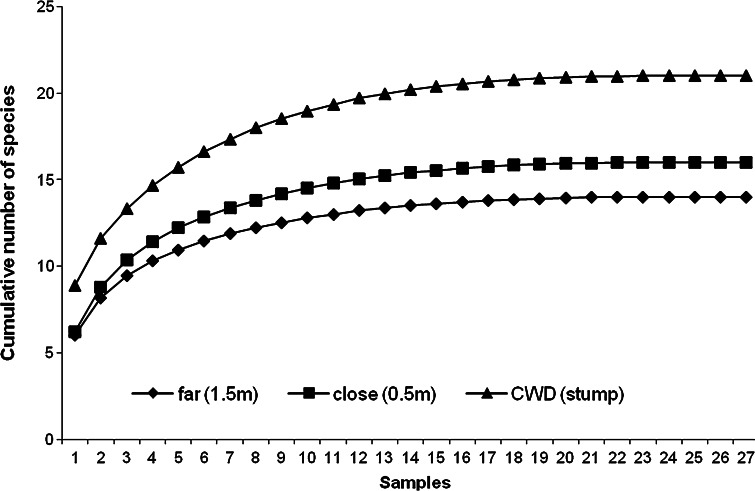
Species accumulation curves in coarse woody debris (CWD; stumps) and litter/soil at 0.5 (*close*) or 1.5 (*far*) m distance from the stumps in boreal Scots pine forests

Species accumulation curves for all microhabitats showed decreased rates of species accrual with increased sampling effort. Species richness (as cumulative number of collected species) in CWD and soil/litter stabilized before 24 samples, reaching 21 species in CWD, 16 at close distance and 14 at far distance (Fig. 2). Species accumulation curves for soil/litter at close and far distances were very similar.

The theoretical species richness, using first- and second-order Jackknife as well as Chao 1 and 2 estimators, were higher in CWD and decreased with increasing distance to the stumps. For instance, the Chao 2 richness estimator (based on presence/absence data) gave 22.19 ± 4.89 species for CWD, 15.83 ± 1.62 species for litter at close distance and 14.22 ± 1.96 species for litter at far distance—all very similar to the numbers actually observed (Table 3).

**Table 3:** Observed and theoretical total mesostigmatid mite species richness for coarse woody debris (CWD) in decayed Scots pine tree stumps and soil/litter at 0.5 and 1.5 m from the stumps

	CWD (stump)	Close (0.5 m)	Far (1.5 m)
Observed	21	16	14
Chao 1 (quantitative)	19.97 ± 2.17	14.93 ± 1.93	13.41 ± 1.95
Chao 2 (presence/absence)	22.19 ± 4.89	15.83 ± 1.62	14.22 ± 1.96
Jack 1st order	20.76 ± 3.16	15.94 ± 2.49	13.79 ± 1.97
Jack 2nd order	20.28 ± 4.59	15.59 ± 3.58	13.45 ± 2.92

### Species assemblages

Five species were restricted in distribution to CWD: *Proctolaelaps fisheri*, *Sejus togatus*, *Trachytes pauperior,* *Vulgarogamasus* sp. and *Zercon curiosus* (“Appendix 1”). In total, the most common mesostigmatid mite species were *Veigaia nemorensis*, *Parazercon radiatus* and *Zercon zelawaiensis*, which represented the majority of the local mesostigmatid mite community (“Appendix 1”).

**Fig. 3:**
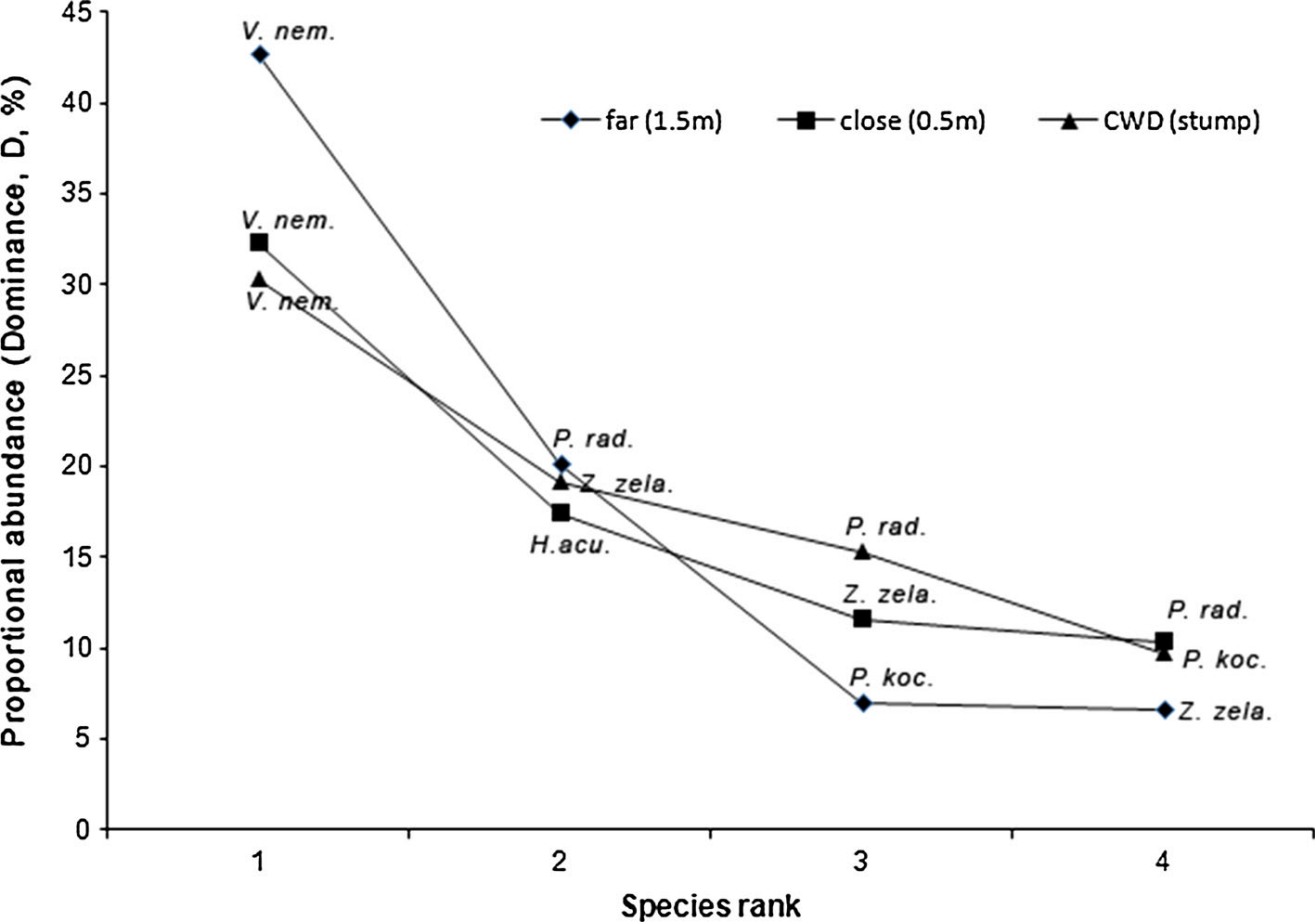
Species rank for mesostigmatid mites in Scots pine stumps (i.e., coarse woody debris, CWD) and litter/soil at 0.5 (close) or 1.5 (*far*) m distance from the stumps. Species names: *V. nem.* = *Veigaia nemorensis*, *H. acu*. = *Hypoaspis aculeifer*, *P. rad.* = *Parazercon radiatus*, *P. koc.* = *Prozercon kochi*, *Z. zela.* = *Zercon zelawaiensis*

Analysis of species ranks revealed differences in the proportional abundance of the dominant species (Fig. 3). The highest value was recorded for *V. nemorensis* in litter samples collected at far distance (1.5 m) from the stump (*D* = 42.7). Slightly lower values were obtained from CWD (*D* = 30.3) and litter at close distance (*D* = 32.3). The proportional abundance appears to decrease most rapidly in litter samples at 1.5 m from the stump and least rapidly in CWD samples (Fig. 3).

**Fig. 4:**
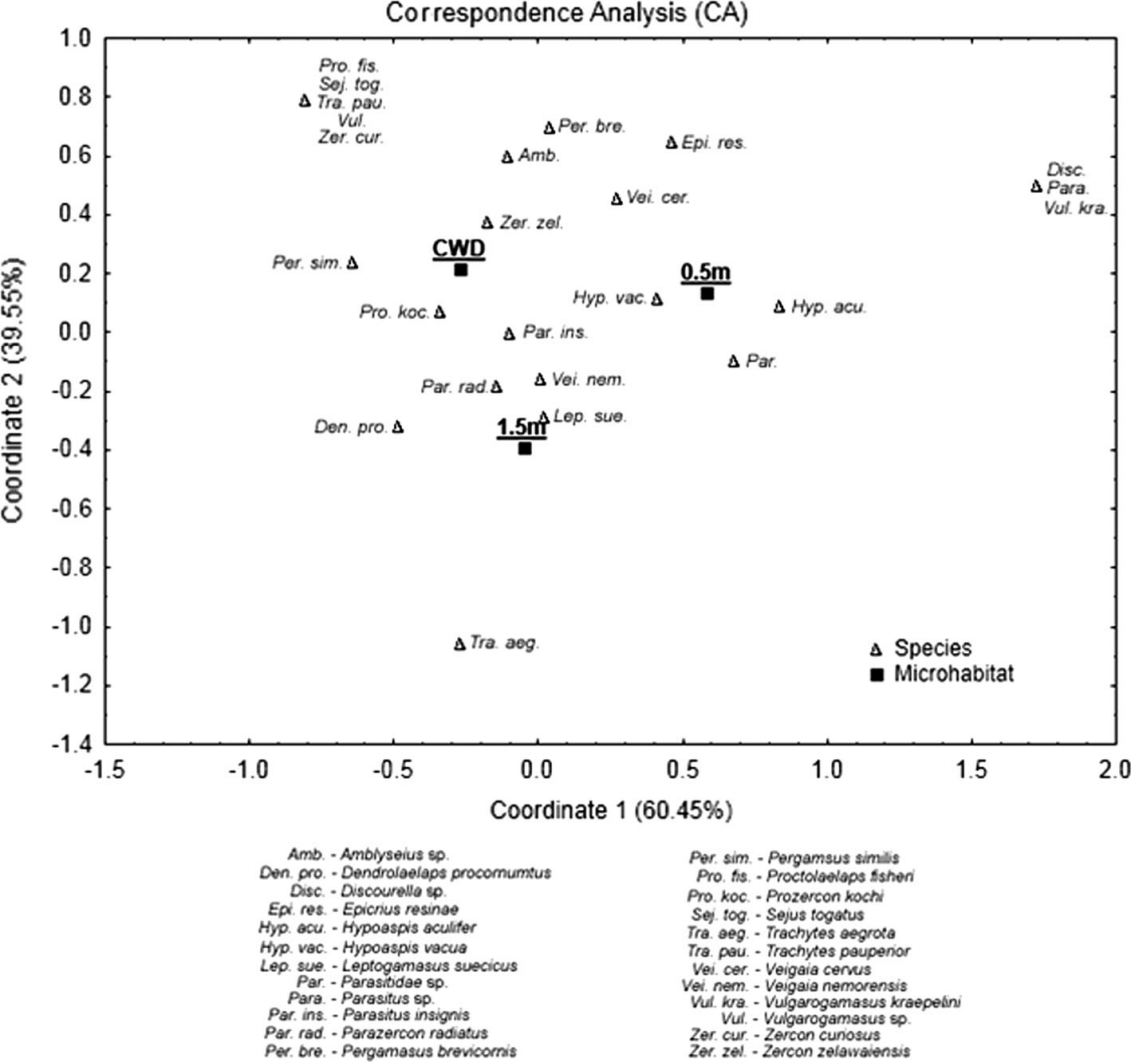
A plot of the first two axes of a correspondence analysis of 24 species and three microhabitats: CWD (coarse woody debris; Scots pine tree stump), 0.5 m (litter/soil at 0.5 m distance from the stump), and 1.5 m (litter/soil at 1.5 m distance from the stump)

In the correspondence analysis (CA) the eigenvalues were not significant and relatively low for axis 1 (λ = 0.11) and axis 2 (λ = 0.07) (Fig. 4). Ordination axes are considered significant if their eigenvalue is higher than 0.3 (Dekkers et al. 1994). Moreover, 100 % of the variance was explained by the first two axes (60.5 and 39.5 %) and the sites are well separated by the ordination plot. Axis 2 appears to divide the communities of CWD and litter at close distance (0.5 m) from that of litter at far distance (1.5 m), whereas axis 2 separates the community of CWD from that of litter at close distance.

## Discussion

Our study indicated that CWD is characterized by higher abundance and diversity of mesostigmatid mites than (nearby) soil/litter. This is in line with studies of Skubała and Sokołowska (2006), who compared oribatid communities between spruce logs and soil/litter, and Skubała and Duras (2008), who investigated oribatids in beech logs and litter—both studies recorded higher abundance and species richness in decaying logs than in the forest floor. Additionally, a recent study of Skubała and Gargul (2011) reported a higher abundance of oribatids and mesostigmatids in CWD (tree hollows) than in the floor in fir-beech forests. Siira-Pietikäinen et al. (2008) documented a three-fold higher abundance of oribatid mites in CWD than in soil/litter in coniferous forests, but equal abundances in deciduous forests. Some studies indicated that dead wood is a poorer substrate for mites than forest floor (Seastedt et al. 1989; Johnston and Crossley 1993).

The impact of distance to CWD on density of selected groups of invertebrates was investigated in a variety of forests, such as red and silver beech (*Notrofagus fusca* and *N. menziesii*) in New Zealand (Evans et al. 2003), oak-beech (*Fagus sylvatica*—*Quercus petrea*) in Germany (Jabin et al. 2004), sugar maple (*Acer saccharum*) in Canada (Varadi-Szabo and Buddle 2006), loblolly pine (*Pinus taeda*) (Ulyshen and Hanula 2009) and oak-maple-hickory (Castro and Wise 2010) in USA. These studies differ in various aspects—for instance, location, forest type, investigated taxonomic groups, type of CWD and distance to CWD—which makes direct comparison of these studies difficult. Generally, these studies assess the impact of very close distance from decayed logs (ca. 0.1 m) relative to far distance (ca. 5 m) (Jabin et al. 2004). Compared to these other studies, the current study revealed the effect of pine stumps (22–25 cm diameter) at the closer distances (0.5–1.5 m). Still, our species richness analysis indicated that the ‘woody island’ stump is characterized by higher species abundance and diversity than soil/litter.

Our research indicated that with increasing distance to CWD, the total number of mite species in the soil/litter matrix decreases. This result is similar to that of Jabin et al. (2004), who reported higher densities of Isopoda, Chilopoda and Pseudoscorpionida in close distance to moderately decayed logs, and that of Castro and Wise (2010), who found a higher density of forest-floor spiders in litter adjacent to CWD. On the other hand, some parameters in our study, such as mean number of species, evenness and Shannon index, did not differ significantly between close and the far distance, although both differ from CWD. This can be explained by our sampling method and the high homogeneity of the forest floor. Compared to other studies, our ‘close distance’ samples were relatively distant—we applied 0.5 m for close distance versus 0.1 m in studies of Jabin et al. (2004) or Kappes et al. (2006). Recent studies of Kappes et al. (2006), investigating litter at (very) close distance (0.1 m) from CWD, indicated that some parameters, such as pH, nutrients and litter accumulation, are higher in litter adjacent to CWD, possibly influencing the abundance and diversity of litter-dwelling snails (Mollusca: Gastropoda). The wide variability in possible indirect effects of CWD on the fauna of surrounding litter may reflect differences in responses of the various arthropod groups (Evans et al. 2003).

Some species, such as *P. fisheri*, *S. togatus*, *T. pauperior* and *Z. curiosus*, were recorded from stumps exclusively (“Appendix 1”). The correspondence analysis indicated that those species characterize the mite community in the CWD microhabitat. Generally, *Trachytes aegrota*, *P. fisheri* and *S. togatus* are known to occur in decayed wood (Karg 1993). The mite communities were dominated by the same main species, i.e. *V. nemorensis*, *P. radiatus* and *Z. zelawaiensis*. This is in contrast to Siira-Pietikäinen et al. (2008) who found different oribatid mites communities in decaying logs versus litter: many of the dominant species in decaying wood were found only rarely in the forest floor. A possible explanation is that some oribatid mites use CWD exclusively and that fallen logs are a refuge for mites normally occurring in forest litter (Skubała and Duras 2008). Surprisingly, we have also found few heteromorphic males of *Hypoaspis (Cosmolaelaps) vacua* which were described from soil samples (Gwiazdowicz 2004).

## Appendix 1

See Table 4.

**Table 4:** Checklist of mesostigmatid mites in coarse woody debris (CWD) in decayed Scots pine tree stumps and soil/litter at 0.5 and 1.5 m from the stumps

No.	Species	Microhabitat					
		CWD (stump)		Close (0.5 m)		Far (1.5 m)	
		*D* (%)	*F* (%)	*D* (%)	*F* (%)	*D* (%)	*F* (%)
1	*Amblyseius* sp.	4.53	88.9 (9)	3.23	44.4 (6)	0.47	11.1 (3)
2	*Dendrolaelaps procornutus*	0.35	11.1 (3)	–	–	0.47	11.1 (3)
3	*Discourella* sp.	–	–	0.65	11.1 (3)	–	–
4	*Epicrius resinae*	0.35	11.1 (3)	0.65	11.1 (3)	–	–
5	*Hypoaspis aculeifer*	2.79	55.6 (9)	17.42	66.7 (8)	5.16	66.7 (9)
6	*Hypoaspis vacua*	1.39	44.4 (7)	3.23	22.2 (8)	1.41	11.1 (3)
7	*Leptogamasus suecicus*	2.79	55.6 (9)	3.23	55.6 (9)	5.16	44.4 (8)
8	*Parasitidae*	0.70	11.1 (3)	3.87	22.2 (3)	1.88	33.3 (6)
9	*Parasitus insignis*	5.92	100 (9)	4.52	44.4 (9)	5.63	55.6 (8)
10	*Parasitus* sp.	–	–	1.94	11.1 (3)	–	–
11	*Parazercon radiatus*	15.33	88.9 (9)	10.32	88.9 (9)	20.19	100 (9)
12	*Pergamasus brevicornis*	0.70	22.2 (3)	0.65	11.1 (2)	–	–
13	*Pergamasus similis*	1.05	22.2 (4)	–	–	0.47	11.1 (3)
14	*Proctolaelaps fisheri*	0.35	11.1 (2)	–	–	–	–
15	*Prozercon kochi*	9.76	88.9 (9)	3.23	44.4 (9)	7.04	66.7 (9)
16	*Sejus togatus*	0.70	22.2 (3)	–	–	–	–
17	*Trachytes aegrota*	0.35	11.1 (3)	–	–	2.35	22.2 (6)
18	*Trachytes pauperior*	0.35	11.1 (3)	–	–	–	–
19	*Veigaia cervus*	1.74	11.1 (5)	2.58	11.1 (3)	0.47	11.1 (3)
20	*Veigaia nemorensis*	30.31	100 (9)	32.26	100 (9)	42.72	100 (9)
21	*Vulgarogamasus kraepelini*	–	–	0.65	11.1 (4)	–	–
22	*Vulgarogamasus* sp.	0.35	11.1 (3)	–	–	–	–
23	*Zercon curiosus*	1.05	22.2 (6)	–	–	–	–
24	*Zercon zelawaiensis*	19.16	88.9 (9)	11.61	66.7 (9)	6.57	55.6 (6)
	Total	100	–	100	–	100	–

Dominance (*D*)—percentage of individuals of the same species, Frequency (*F*)—percentage of samples in which a species occurred. Number of stumps in which the species occur is added in parentheses (maximum 9 stumps)
